# Homeopathy a Applied to Veterinary Medicine*Read before the Massachusetts Veterinary Association, December, 1893.

**Published:** 1894-03

**Authors:** F. B. Carlton


### HOMEOPATHY AS APPLIED TO VETERINARY MEDICINE.*

By F. B. Carlton, M.D.V.

When I received a note from our Secretary reminding me that a paper was expected from me to-night, I had forgotten that twelve months ago I had promised one, consequently I had made no attempt at preparing it. So on the plea of lack of time, and the fact that the subject was not of my own choosing, but suggested by Dr. Peters, I ask you to excuse my shortcomings, and perhaps you may get an idea or two from what I am to read, the subject having at least the recommendation of novelty.

As some of you are aware, about fifteen months ago I gave up my veterinary practice in order to study human medicine, and desiring some knowledge of homeopathy, I entered the Boston University School of Medicine, as a candidate for the degree of M.D. What little I have to say will be largely taken from the work of others, my own experience necessarily having been rather limited.

At first let me give briefly the history of homeopathy, and the principle which underlies the application of medicines under the law of *similia similibus curantur*.

The founder of the school was Samuel Hahnemann, a German physician, who late in the eighteenth century, while experimenting with cinchona bark, discovered its power to produce certain symptoms similar to those existing in diseases for whose cure it was administered. The idea that he had discovered a universal law flashed upon him, and he proceeded to "prove" various other substances, and to bring into prominence medicines whose therapeutic virtues had not previously been known.

As you are doubtless aware, "proving" substance consists in administering to a number of healthy persons some substances the nature of which is to them unknown, and noting the effect produced. It is customary to give one or more of the provers some innocuous substance, in order that symptoms due to the imagination may be eliminated.

Under the law of similars, the remedy would be useful in the cure of a disease the symptoms of which resembled those produced in the provers.

Remember now that homeopathy does not necessarily imply infinitesimal doses, although a good many of that school do believe that they can effect cures with quantities of drugs so small that

* Read before the Massachusetts Veterinary Association, December, 1893.

our imagination cannot reach them. Be that as it may, the homeopathic idea is not large dose of a remedy, not small dose, but the right remedy.

And now we come to the brief discussions of a number of remedies : Colocynth, the bitter cucumber, as many of you will testify, has in its proving the following symptoms : intermittent, colicky and increasing spasmodic pains, accompanied by a cold sweat, and relieved by hard pressure, very severe while the paroxysms last, but the intermissions are free from pain. Colocynth will almost immediately relieve a case with the above symptoms, as I have demonstrated to my own satisfaction in several cases of spasmodic colic. The pain should be purely a neuralgic one, and not due to any organic lesion, in order to have colocynth of avail.

Aloes, with which you are all familiar as a cathartic, is also useful in cases of diarrhœa not produced by the drug. A case of scouring in which there is some rumbling of the bowels, pain before passage of fæces, which escape without effort and accompanied by a good deal of flatus, and more or less straining when the rectum is empty, such a case would probably yield to aloes. I can personally recommend aloes in hæmorrhoids, both in man and beast. It will be found useful in various inflammations of the large intestines, and especially in congestive diseases of the anus and rectum.

Nux vomica, which, in its provings, was found to produce constipation, gastralgia and dyspepsia as a few of its symptoms, is an exceedingly important member of the homeopathic materia medica. Unfortunately for us, it acts more powerfully on the carnivora and omnivora than on the herbivorous animals, still it is by no means without value in veterinary practice. It is of use in colic from over-eating, accompanied by constipation and frequent ineffectual attempts to pass manure. It is of value in dogs who are over-fed, constipated and have insufficient exercise, but remember that no inflammation of the mucous surface should exist, if it is desired to use nux.

This is one of the many remedies that is used homeopathically by the old school, who prescribe it, or its alkaloid strychnia, in small doses in sick headache or migraine, cardialgia and gastralgia.

In my own experience it has seemed indicated in the following: In a case of torpidity of the bowel of a horse. 2. In a so-called fitty horse, whose trouble appeared to me to be caused by indigestion. 3 and 4. In two cases of gastric disturbance in dogs, both accompanied by constipation, fever and want of appetite, and one of which was rather severe.

In all of the above the treatment was eminently successful, the administration of nux vomica being followed by recovery, and that rather soon. Of course the animals might have recovered anyhow, but that is something one might say about any case and whatever the treatment, excepting of course in surgery.

Belladonna is well known to you. That again is a drug which doubtless you all have used homeopathically. Take one instance only, that of pharyngitis. Belladonna has in its proving the same inflamed, dry, sore throat for which we are in the habit of prescribing it. In conjunctivitis and various other inflammations of the eye it is widely used.

Its sphere is almost unlimited, and it would be useless for me to do more than mention it. In one case of a horse subject to fits, as the owner said, I used it for two weeks, reducing the number of attacks from one or more daily to one during the fortnight. Unfortunately I left the place then, and am unable to state whether a permanent cure resulted.

In the *Medical Advance* for October, 1893, are reported a number of cases from which I shall quote, in all of which the treatment was successful.

Rhus toxicodendron, the sumach or poison oak, was used in case of a horse lame forward. Conium, or hemlock, was used in several cases of azoturia. As far as I can find out, conium is homeopathic to the paralysis of the disease. As azoturia may, and often does, result fatally, it strikes me that conium is well worth a trial

Pneumonia with phosphorus for a remedy. Glonoin, or nitroglycerin, was given to a horse with blind staggers.

Kali. carb., corbonate of potash, in leucorrhœa in bitch with general inflammation of the genitalia.

Arsenic in diarrhœa in an ox and in chicken cholera.

What I have said will be a nucleus on which to build to those of you who are at all interested in the subject. Remember that I have merely touched on a few drugs which are in daily use by homeopathists. My knowledge of the subject is very much limited, but the more time I give to it the more I am convinced that on the whole it is the most rational system of medicine known at the present day. I can only say, don't condemn it until you have given it careful study and trial. I was a few years ago as sceptical in regard to homeopathy as are any of you, but reflection, study and actual practice have convinced me that curable diseases can be cured more easily and quickly by a rational homeopathy than by any other known method. And if this be true of men, why not apply the principle to veterinary practice?

